# Central nervous system metastases in metastatic breast cancer: Incidence, subtype-specific patterns, and outcomes from the Austrian AGMT_MBC registry

**DOI:** 10.1016/j.breast.2026.104891

**Published:** 2026-08-03

**Authors:** Simon Peter Gampenrieder, Vanessa Castagnaviz, Rupert Bartsch, Thamer Sliwa, Angelika Pichler, Renate Pusch, Clemens Dormann, Christoph Suppan, Sonja Heibl, Lukas Scagnetti, Margit Sandholzer, Thomas Winder, August Felix Zabernigg, Clemens Schmitt, Daniel Egle, Christopher Hager, Petra Pichler, Florian Roitner, Johannes Andel, Kathrin Strasser-Weippl, Michael Hubalek, Michael Knauer, Christian Fridolin Singer, Richard Greil, Gabriel Rinnerthaler

**Affiliations:** a Department of Internal Medicine III with Haematology, Medical Oncology, Haemostaseology, Infectiology and Rheumatology, Oncologic Center, Paracelsus Medical University Salzburg, Salzburg, Austria; b Cancer Cluster Salzburg, Salzburg, Austria; c Austrian Group for Medical Tumor Therapy (AGMT) GmbH, Salzburg, Austria; d Department of Medicine I, Division of Oncology, Medical University of Vienna, Vienna, Austria; e Internal Medicine - Department for Haemato-Oncology, LKH Hochsteiermark, Leoben, Austria; f Internal Medicine I for Medical Oncology and Hematology, Ordensklinikum Linz - Barmherzige Schwestern, Linz, Austria; g Division of Oncology, Department for Internal Medicine, Medical University Graz, Graz, Austria; h Department of Internal Medicine IV, Klinikum Wels-Grieskirchen GmbH, Wels, Austria; i Department of Internal Medicine II, Academic Teaching Hospital Feldkirch, Feldkirch, Austria; j Department of Internal Medicine, County Hospital Kufstein, Kufstein, Austria; k Department for Hematology and Internal Oncology, Med Campus III, Kepler University Hospital Linz, Johannes Kepler Universität Linz, Linz, Austria; l Department of Gynaecology, Medical University Innsbruck, Innsbruck, Austria; m Breast Center Dornbirn, Dornbirn, Austria; n Department for Internal Medicine 1, University Hospital St.Pölten, St. Pölten, Austria; o Department of Internal Medicine II, Hospital St. Josef, Braunau, Austria; p Department of Internal Medicine II, Pyhrn-Eisenwurzen Klinikum Steyr, Steyr, Austria; q Department of Gynecology, Breast Health Center Schwaz, Schwaz, Austria; r Tumor and Breast Center Eastern Switzerland, St. Gallen, Switzerland; s Department of Obstetrics and Gynecology and Comprehensive Cancer Center, Medical University of Vienna, Vienna, Austria; t Salzburg Cancer Research Institute-Center for Clinical Cancer and Immunology Trials (SCRI-CCCIT) GmbH, Salzburg, Austria; u Salzburg Cancer Research Institute-Laboratory for Immunological and Molecular Cancer Research (SCRI-LIMCR), Salzburg, Austria

**Keywords:** Brain metastases, Luminal, HER2-positive, Triple-negative, Real-world data

## Abstract

**Background:**

Brain metastases (BM) cause substantial morbidity and reduced survival in metastatic breast cancer (MBC). Therapeutic advances may have altered BM incidence and outcomes across subtypes. Real-world data are crucial to assess these trends and guide BM-specific clinical trials. Here, we report BM data from the Austrian Study Group for Medical Tumor Therapy MBC registry.

**Methods:**

Patients with known hormone receptor (HR) and HER2 status and sufficient outcome data were included. Logistic regression was used to assess the risk of BM. Overall survival (OS) was estimated using Kaplan–Meier methods and compared by log-rank tests. Multivariable Cox models with BM as a time-dependent variable and competing-risk analyses were applied.

**Results:**

Among 2775 patients, 477 (17.2%) developed BM during the metastatic course; 56 patients (2.0%) had brain-only disease at first metastatic diagnosis. Compared with luminal MBC, BM risk was higher in HR-/HER2+ (OR 3.57), HR+/HER2+ (OR 4.43), and triple-negative breast cancer (TNBC; OR 2.91). At a median follow-up of 77 months, BM were associated with significantly shorter OS (HR 4.23; p < 0.001). TNBC showed the shortest BM-free survival (7.1 months) and poorest OS after BM diagnosis (4.7 months). Brain-only disease was associated with improved OS compared to concurrent extracranial disease (HR 0.58; p = 0.001). Use of whole brain radiotherapy declined over time, while neurosurgery or focal radiotherapy were associated with longer OS. Receptor discordance included HR loss (14.5%) and HER2 gain (9.8%).

**Conclusion:**

BM remain frequent and prognostically relevant in MBC, underscoring the need for improved risk stratification and subtype-specific therapies.

## Highlights

• Brain metastases remain frequent and confer a major survival disadvantage.
• Prognosis differs markedly by breast cancer subtype and disease distribution.
• Real-world data reveal unmet needs beyond established therapies.

## Graphical abstract

**Figure:**
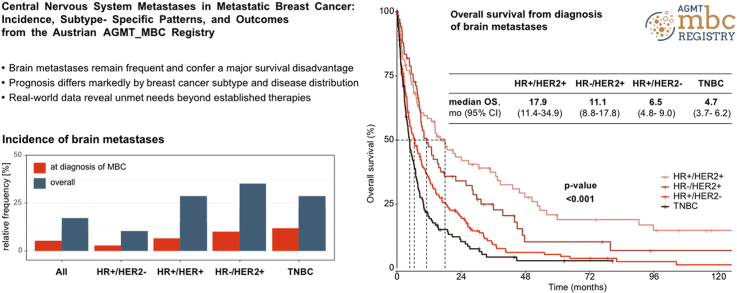

## 1 Introduction

Breast cancer (BC) brain metastases (BM) are associated with increased morbidity, neurologic impairment, diminished quality-of-life (QoL), and reduced overall survival (OS) [1]. Because the blood-brain-barrier (BBB) limits the efficacy of systemic therapy in BM, locoregional treatments such as surgery and radiotherapy (including stereotactic radiotherapy, radiosurgery, and whole brain radiation therapy [WBRT]), are used to achieve local disease control. WBRT, however, results in early QoL reduction and neurocognitive decline [2]. Therefore, the interest in systemic therapies for BM providing bicompartmental disease control and avoiding WBRT has increased. An intact BBB prevents adequate drug penetration of conventional chemotherapies and antibodies into the brain parenchyma, making the central nervous system (CNS) a sanctuary site for cancer cells. Tumors however disrupt BBB integrity, leading to the formation of a specialized neurovascular unit known as the brain-tumor barrier (BTB), characterized by uneven permeability and active efflux of molecules [3,4]. While these facts may still result in varying drug concentrations [5], the increased permeability of the BTB serves as a rational for systemic therapy approaches and this concept has been proven successful in HER2-positive BC so far. In contrast, systemic therapy of BM is ill established beyond the HER2-positive subtype and little information is available regarding the potential intracranial activity of novel agents as patients with BM, particularly those with active BM (newly diagnosed or progressing after locoregional treatment), are often explicitly excluded from clinical trials. Moreover, in the absence of routine screening for asymptomatic BM, their true incidence is likely underestimated, further complicating clinical assessment and trial design.

In the absence of robust evidence from randomized studies, real-world data (RWD) play a critical role in evaluating and quantifying clinical scenarios in the management of BM and may also serve as backbone for clinical trial development. Here, we present BM data from the national Austrian metastatic breast cancer (MBC) registry led by the Austrian Group for Medical Tumor Therapy (AGMT).

## 2 Patients and methods

The AGMT_MBC Registry is an ongoing, multicenter registry for MBC patients in Austria. For this analysis, patients with available hormone receptor (HR) and HER2 status, as well as sufficient outcome data, were included. In case of BM diagnosis, the date of diagnosis was required.

For investigation of group differences following tests were applied: Wilcoxon rank sum test for metric variables; Chi-Square/Fisher's exact test for ordinal/nominal variables. Unadjusted, univariable OS probabilities were calculated using the Kaplan-Meier method and compared with the log-rank test. Multivariable adjusted hazard ratios (HR) were estimated using Cox regression models. When applicable, diagnosis of BM was considered as a time-dependent variable. Logistic regression was conducted to examine the association of prespecified covariables and developing BM. The multivariable analyses incorporated the following parameters: breast cancer subtype (HR+/HER2-vs. HR + HER2+ vs. HR-/HER2+ vs. TNBC), age at diagnosis of metastatic disease (as a continuous variable, with interaction terms for menopausal status in the Cox regression), disease-free survival (*de novo* metastatic or ≥24 months vs. <24 months), presence of visceral disease (yes vs. no), and the number of metastatic sites at the time of metastatic disease diagnosis (1 vs. 2-3 vs. ≥4). Competing risk analysis was performed to investigate the cumulative incidence of BM over time for covariables CDK4/6 inhibitor in first line (yes vs. no; HR+/HER2-subgroup) and Pertuzumab in first line (yes. vs. no; HER2+ subgroup) with death as competing event. To assess temporal changes in the overall incidence of BM, patients were grouped into three periods according to the date of metastatic disease diagnosis (2005–2011, 2012–2018, and 2019–2025). For each period, the 5-year overall incidence was calculated as the sum of the baseline prevalence of BM (present at metastatic diagnosis) and the 5-year cumulative incidence of BM during follow-up. To analyze the outcome under current treatment standards, which have significantly improved the prognosis, following intervals were selected for analysis based on the availability of the substances, the following intervals were selected for analysis: 2017-2022 (CDK4/6 inhibitor) and 2014-2022 (Pertuzumab). When applicable, results were presented along with 95% confidence intervals (95%CI). Statistical tests were carried out at 5% significance level. P-values in multiple models were corrected for multiple testing using Bonferroni-Holm. All calculations were done using statistical software R (version 4.5.1).

## 3 Results

### 3.1 Incidence of brain metastasis

As of 11th March 2025, a total of 2956 patients were included in the registry. Among 2775 evaluable patients, 477 (17.2%) had documented BM. Compared to patients without BM, those with BM were younger at the time of metastatic disease diagnosis, more frequently had HR-negative and/or HER2-positive tumors, were less likely to have *de novo* metastatic disease, and had shorter disease-free survival (DFS). Moreover, patients with BM more often presented with visceral (non-CNS) involvement at the time of metastatic disease diagnosis (52.6% vs. 43.3%) and with leptomeningeal disease (11.5% vs. 3.0%, respectively). Detailed patient characteristics are summarized in Table S1 in the Supplement.

The median number of systemic therapy lines administered before and after the diagnosis of BM was 1 (range 0-8) and 1 (range 0-10), respectively. The median total treatment number was higher in patients with BM (3 [range 0-14] vs. 2 [range 0-14]). Anti-HER2 antibody drug conjugates (ADCs), anti-HER2 tyrosine kinase inhibitors (TKIs) and PARP inhibitors were used more frequently in patients with BM. CDK4/6 inhibitors, conversely, were adopted less frequently in patients with BM than in those without BM (descriptive only) (Table S2 in the Supplement).

**Fig. 1:**
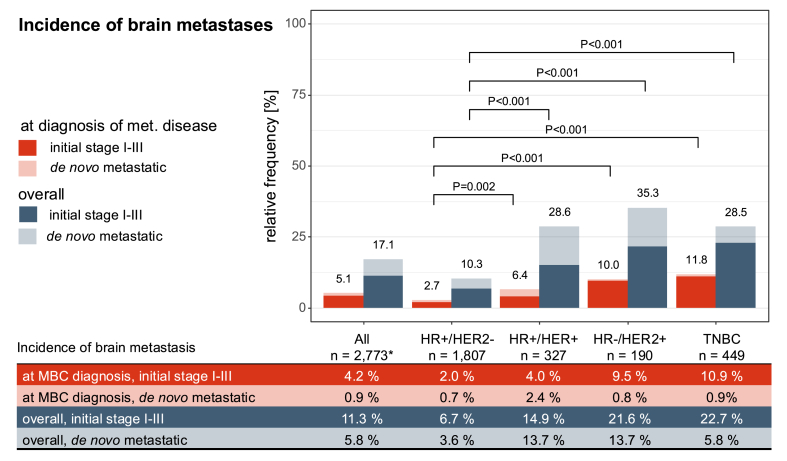
Incidence of brain metastasis at diagnosis of metastatic disease and during the metastatic course. *2 patients in HR+/HER + subgroup excluded due to unknown initial stage.

The incidence of BM at the time of primary diagnosis of metastatic disease, as well as the cumulative incidence during the metastatic course, were significantly higher in HR-/HER2+ tumors (10.0% [19/190] and 35.3% [67/190], respectively), HR+/HER2+ tumors (6.7% [22/329] and 29.2% [96/329], respectively) and triple-negative tumors (11.8% [53/449] and 28.5% [128/449], respectively) compared to luminal tumors (2.7% [49/1807] and 10.3% [168/1807], respectively). All *post-hoc* Chi-Square tests for these comparisons were highly statistically significant (Fig. 1).

Temporal changes in overall incidence were assessed by calculating the 5-year overall incidence - defined as the sum of baseline prevalence and 5-year cumulative incidence during follow-up - across three metastatic diagnosis periods (2005–2011, 2012–2018, and 2019–2025). Overall, the 5-year incidence of brain metastases increased from 7.9% in 2005–2011 to 11.1% in 2012–2018 and remained elevated at 10.3% in 2019–2025. This increase was primarily driven by the HR+/HER2+ and TNBC subtypes. Across all periods, the highest incidence was consistently observed in HR+/HER2+ and TNBC subtypes (Table S3 in the Supplement).

In multivariable logistic regression, in addition to HER2-positive and triple-negative subtype, younger age at diagnosis of MBC, shorter DFS, and the presence of ≥4 metastatic sites at diagnosis were statistically significantly associated with the occurrence of BM (Table 1).

**Table 1:** Multivariable logistic regression analysis for associations with BM (n = 2613).

Association with BM	OR (95%-CI)	*p*
**Breast cancer subtype**		
*HR-/HER2+ vs. HR+/HER2-*	4.43 (3.08-6.32)	**<0.001**
*HR+/HER2+ vs. HR+/HER2-*	3.57 (2.65-4.81)	**<0.001**
*TNBC vs. HR+/HER2-*	2.91 (2.18-3.86)	**<0.001**
**Age**^a^ (continuous)	0.97 (0.96-0.98)	**<0.001**
**Disease-free survival (DFS)**		
*≥* 24 months *or de novo vs. <* 24 months	0.69 (0.53-0.90)	**0.022**
**Extracranial visceral disease**^a^		
*yes vs. no*	1.01 (0.79-1.30)	1.000
**Number of metastatic sites**^a^		
*2-3 vs. 1*	0.96 (0.75-1.24)	1.000
*≥ 4 vs. 1*	2.15 (1.45-3.18)	**0.001**

a At diagnosis of metastatic disease.

### 3.2 Brain metastasis-free survival (BMFS)

BMFS, measured from the date of metastatic disease diagnosis, was 12.4 months (95% CI 11.0-14.8) in the overall BM population. However, substantial differences were observed between BC subtypes with longer BMFS for ER positive subtypes: 16.4 months in HR+/HER2+, 17.9 months in HR+/HER2-, 11.0 months in HR-/HER2+ and only 7.1 months in triple-negative disease (Table S4 in the Supplement).

### 3.3 Overall survival

**Fig. 2:**
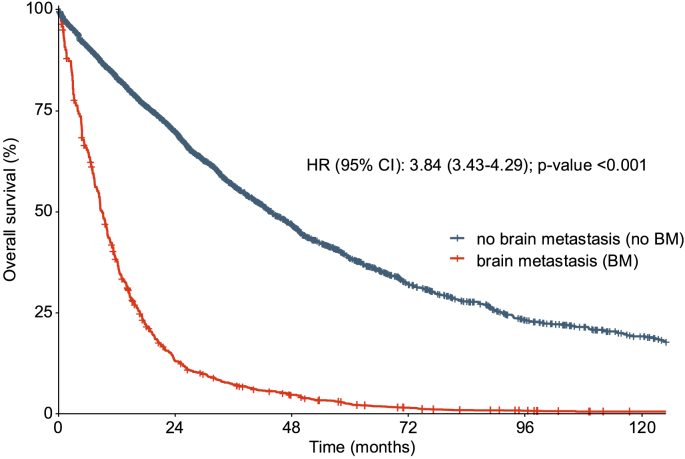
Adjusted overall survival estimates* comparing patients with and without brain metastases. * from diagnosis of MBC, estimated from an univariable time-dependent Cox model.

At a median follow-up of 77.0 months (95%-CI 73.1-83.2), patients who developed BM had a significantly shorter OS (calculated from diagnosis of MBC) compared to patients without BM, both in time-dependent univariable (HR 3.84; 95%CI 3.43-4.29; P < 0.001; Fig. 2) and multivariable analyses (HR 4.23; 95%CI 3.73-4.81; P < 0.001, Table S5). The unadjusted median OS was 26.2 (95%CI 23.5-30.9) months for patients with BM and 39.6 (95%CI 36.8-42.1) months for patients without BM.

**Fig. 3:**
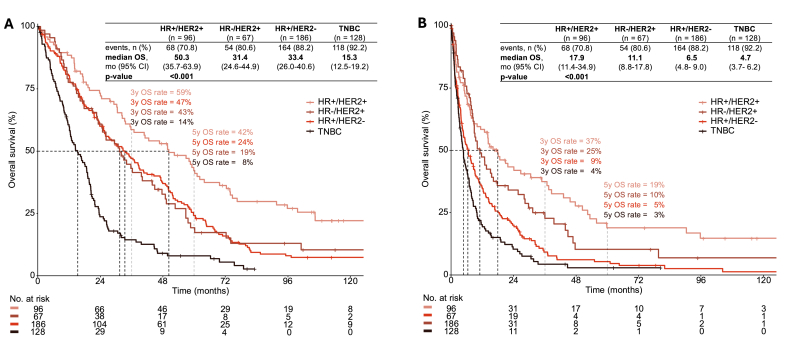
Overall survival estimates in patients with brain metastases according to breast cancer subtype. **Legend:** OS estimated of patients with BM according to molecular subtype: (A) from diagnosis of MBC (B) from diagnosis of BM.

OS in patients with BM differed significantly between breast cancer subtypes, with a median OS of 33.4 months (95%CI 26.0-40.6), 50.3 months (95%CI 35.7-63.9), 31.4 months (95%CI 24.6-44.9) and 15.3 months (95%CI 12.5-19.2) in HR+/HER2-, HR+/HER2+, HR-/HER2+, and TNBC, respectively (overall log-rank P < 0.001; Fig. 3A). Similarly, OS from BM diagnosis was the shortest in TNBC (4.7 months; 95%CI 3.7-6.2) followed by HR+/HER2- (6.5 months; 95%CI 4.8-9.0), HR-/HER2+ (11.1 months; 95%CI 8.8-17.8) and HR+/HER2+ BC (17.9 months; 95%CI 11.4-34.9) (overall log-rank P < 0.001; Fig. 3B). In the overall cohort, the median OS after diagnosis of BM was 7.5 months (95%CI 6.4-8.6).

### 3.4 Brain only versus concomitant extracranial metastases

In our registry, 56 patients (11.7%; 2.0% of the total population) presented with BM as the first metastatic site, without concurrent extracranial metastases (brain-only disease). Their baseline characteristics differed significantly from patients with concomitant extracranial metastases (n = 421; Table S6). Patients with brain-only disease more often had recurrent disease rather than *de novo* MBC and a shorter DFS (<24 months). In addition, they were more often diagnosed with HR-/HER2+ and less frequently with HR+/HER2-disease (Table S6). OS after diagnosis of BM was 12.9 months in patients with brain-only disease, which was significantly longer compared with patients presenting with concurrent extracranial metastases (median OS 7.0 months; HR 0.58; 95%CI 0.42-0.80; p = 0.001; Fig. S1).

### 3.5 Discordance of receptor-status between primary tumor and BM

Diagnostic or therapeutic neurosurgery was performed in 75 patients (15.7%) and was more common among those with brain-only disease (28/56; 50.0%). Among patients with available receptor data, the receptor status of the most recent primary tumor and the corresponding BM was concordant in the majority of cases. However, changes in ER and HER2 status were observed in 9 of 55 patients (16.4%) and 7 of 51 patients (13.7%), respectively. The most frequent changes were a loss of ER expression (ER + to ER-, 8/55; 14.5%) and a gain of HER2 expression (HER2-to HER2+, 5/51; 9.8%; Fig. S2).

### 3.6 Focal therapies and their influence on OS

**Fig. 4:**
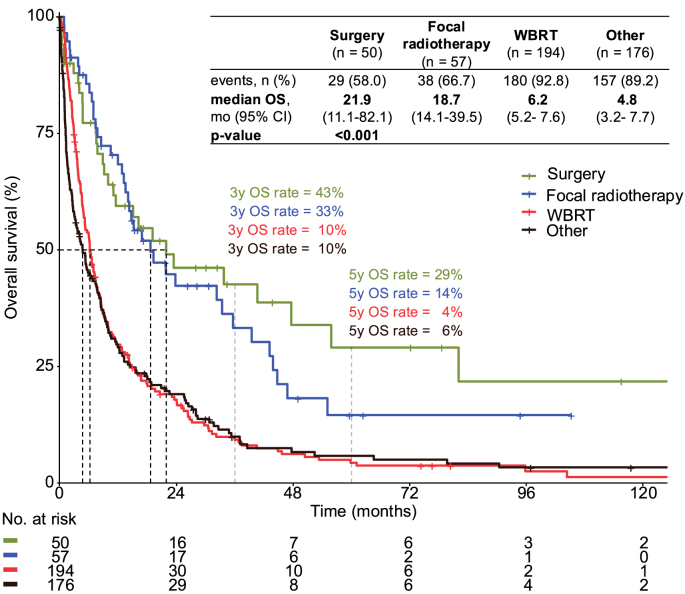
Overall survival after diagnosis of brain metastases according to the type of primary local treatment. **Legend**: If whole brain radiotherapy (WBRT) was initiated within two months of BM diagnosis, patients were categorized as receiving WBRT, irrespective of prior focal radiotherapy or neurosurgery. The category “Other” comprises 23 patients who received WBRT more than two months after diagnosis and 153 patients who did not receive any local therapy for BM.

Most patients with BM (71.9%) received cranial radiotherapy, which consisted of WBRT in 59.8% of patients, focal radiotherapy in 17.8% of patients and both types of radiotherapy in 10.2%. In 12.2% of patients, the type of radiotherapy was unknown. Importantly, the rate of WBRT as the primary treatment after BM diagnosis steadily decreased over time in all BC subtypes with the lowest rates of primary WBRT in the HR-/HER2+ subgroup (Table 2). OS by type of local therapy is shown in Fig. 4. Patients receiving upfront WBRT had the shortest OS compared to patients undergoing neurosurgery or focal radiotherapy: 6.2 months (95%CI 5.2-7.6) vs. 21.9 months (95%CI 11.1-82.1) vs. 18.7 months (95%CI 14.1-39.5). Patients with brain-only disease had a higher probability for neurosurgery as primary local treatment compared to patients with concurrent extracranial metastases (21/56 [38.0%] vs. 29/421 [6.9%]; OR 8.0; 95%CI 4.0-16.4; p < 0.001).

**Table 2:** Use of whole brain radiotherapy (WBRT) as primary local treatment, defined as start of radiotherapy within 2 months of BM diagnosis (regardless of prior focal radiotherapy or neurosurgery) according to the date of BM diagnosis and breast cancer subtype, respectively.

Time interval	All	HR+/HER2-	HR+/HER2+	HR-/HER2+	TNBC
2005-2025	194/477 (**40.7**%)	69/186 (**37.0**%)	41/96 (**41.7**%)	25/67 (**36.2**%)	59/128 (**46.0**%)
2005-2011	43/78 (**55.1%**)	13/27 (**48.1%**)	13/21 (**61.9%**)	12/18 (**66.7%**)	5/12 (**41.7%**)
2012-2018	99/219 (**45.2%**)	35/81 (**43.2%**)	16/38 (**42.1%**)	9/29 (**31.0%**)	39/71 (**54.9%**)
2019-2025	52/180 (**28.9%**)	21/78 (**26.9%**)	12/37 (**32.4%**)	4/20 (**20.0%**)	15/45 (**33.3%**)

### 3.7 Influence of life-prolonging systemic therapies on BM incidence

**Fig. 5:**
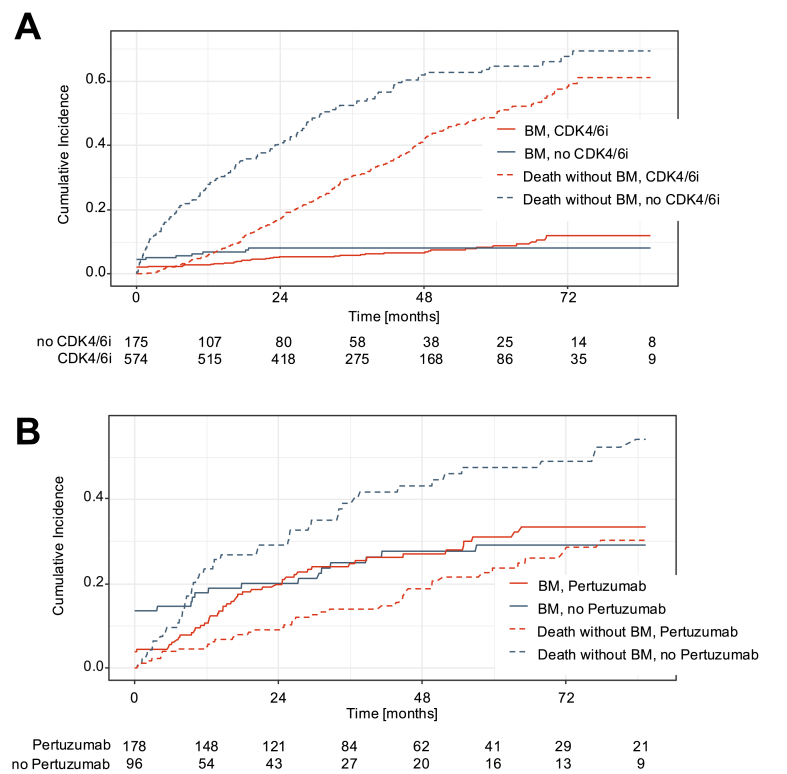
Incidence of brain metastases in patients treated in first-line with or without CDK4/6 inhibitors or pertuzumab **Legend**: Cumulative incidence of BM in patients with HR+/HER2- MBC treated with first-line CDK4/6 inhibitors vs. not (A) and in patients with HER2+ MBC treated with first-line pertuzumab vs. not (B).

To investigate the impact of life-prolonging therapies such as CDK4/6 inhibitors and the anti-HER2 antibody pertuzumab on the incidence of BM, a competing risk analysis was performed. Among patients with HR+/HER2-and HER2+ MBC diagnosed after the availability of these agents in Austria, the risk of developing BM or death was significantly reduced in those receiving first-line CDK4/6 inhibitors or pertuzumab, respectively. When considering the risk of BM only, the cumulative incidence curves for patients with or without CDK4/6 inhibitor or pertuzumab treatment crossed after about four years of follow-up, suggesting that prolonged extracranial disease control may ultimately increase the overall risk of BM (Fig. 5A and B)

## 4 Discussion

In this nation-wide registry including nearly 3000 patients with MBC, with a median follow-up of 77.0 months, BM were documented in 17.2% of patients. The incidence was significantly higher among patients with HR-/HER2+ (35.3%), HR+/HER2+ (29.2%), and triple-negative tumors (28.5%) compared to those with HR+/HER2-tumors (10.3%). Beyond BC subtype, younger age, shorter disease-free survival (<24 months), and high metastatic burden at diagnosis (≥4 metastatic sites) were identified as independent risk factors for BM development. Patients with HR-negative tumors not only had higher BM rates but also had shorter BMFS, with shortest BMFS observed in TNBC patients (7.1 months). The occurrence of BM was associated with markedly reduced OS: patients with BM had a median OS of 9.2 months compared with 43.9 months among those without BM at data cut-off (HR 3.84). Within the BM cohort, patients with HR+/HER2+ disease had the longest OS, whereas OS was shortest in TNBC.

The cumulative incidence of BM observed in our registry is consistent with previously published data. An earlier autopsy study reported a BM incidence of 18.5% [6], while a recent meta-analysis found a pooled cumulative incidence of 15% [7]. In line with our findings, the above mentioned meta-analysis also reported significantly higher BM risks in patients with HER2+ (31%) or TNBC (32%) compared to those with HR+/HER2- MBC (15%) [7]. Since a substantial proportion of patients in our registry (approximately 30%) was still alive at the time of data cut-off, the actual BM incidence may increase further over time. Similar to others [8], we observed an increase in the overall incidence of BM in our cohort after 2005-2011 mainly driven by the triple-negative and HR+/HER2+ subtypes. Notably, fewer patients with TNBC and HR+/HER2+ disease were enrolled in the earliest diagnostic period (2005–2011) compared with subsequent periods, which should be considered when interpreting temporal trends and subtype-specific differences, as these subtypes are associated with a higher risk of brain metastases. Apart from the increase observed between 2005–2011 and 2012–2018 in the two mentioned subtypes, no further increase across diagnostic periods was evident.

Several factors have been proposed that could contribute to an increased incidence of BM: (1) improved systemic therapies prolong survival and thus extend the time at risk for developing BM. This argument is supported by our observation that patients treated with life-prolonging agents, such as CDK4/6 inhibitors or pertuzumab in the first-line setting, not only had a substantially longer OS but also an increased cumulative risk of BM when they lived longer than four years. (2) more sensitive and widely accessible neuroimaging enables earlier detection of asymptomatic or smaller lesions, and (3) increased clinical awareness and vigilance regarding BM risk. Moreover, incidence may continue to rise following the recent update of the EANO/ESMO guidelines [2], which now considers BM screening for patients with HER2+ and TNBC due to the known neurotropic behavior of these subtypes. Incorporating additional BM risk factors for BM – such as young age, short DFS, and high metastatic burden at diagnosis – as identified in our cohort as well as in previous studies [9], could support the development of a more personalized and practical screening strategy in these high-risk populations.

In 1979, DiStefano and colleagues reported a mOS of 4 months in patients with BM, and 1-2 months in patients without intervention [10]. Despite the improvement in both systemic and locoregional treatments, patients with BM still have an unfavorable prognosis compared to those without intracranial disease. The mOS after diagnosis of BM in our cohort was 7.5 months. Not surprisingly, survival of patients with BM differed by BC-subtype, in line with previous reports [11,12]. In our registry, patients with TNBC had the shortest mOS both when calculated from diagnosis of MBC and from diagnosis of BM, while patients with luminal or HER2+ MBC had significantly longer OS. In the intrinsically aggressive HER2+ subtype, this observation is most likely due to the availability of effective systemic treatments such as the selective HER2 TKI tucatinib and ADC trastuzumab deruxtecan (T-DXd) [[13], [14], [15]]. Therefore, current guidelines recommend the administration of both T-DXd (preferred) and the combination of tucatinib, trastuzumab, and capecitabine in patients with active BM [16], thereby offering the chance for delaying WBRT. Unfortunately, data regarding intracranial response rates or duration of CNS responses with these substances were not available in our cohort. To date, no anti-HER2 agent has been conclusively shown to prevent BM in either the neoadjuvant or metastatic setting [[17], [18], [19], [20]]. However, neratinib in the adjuvant ExteNET trial [21] and tucatinib in the metastatic HER2CLIMB trial [22] both demonstrated a reduced risk of CNS recurrence in exploratory analyses. Consequently, the results of the post-neoadjuvant CompassHER2 RD trial investigating T-DM1 in combination with tucatinib (ClinicalTrials.gov Identifier: NCT04457596) are eagerly awaited. In the recently presented DESTINY-Breast05 study, postneoadjuvant treatment with T-DXd reduced CNS recurrences by 30% but short follow-up prevents any definitive conclusions [23]. In HER2-negative breast cancer, substances with proven intracranial activity are scarce, however the PARP inhibitor olaparib reduced the risk for intra- and extracranial metastases to a similar degree in the OlympiA trial [24].

Interestingly, although patients with luminal BC have a lower individual risk of developing BM, their higher overall prevalence means that the majority of patients with BM in our registry had luminal disease (65%). Most patients with luminal MBC develop BM late in the disease course: the incidence of BM at the initial diagnosis of MBC was only 2.7%, and the median time to BM was 17.9 months, after a median of 1 therapy line for MBC (range 1 -8) prior to BM diagnosis. Despite the generally favorable prognosis of luminal MBC, the mOS of 6.5 months following BM diagnosis remains short, reflecting both the limited availability of drugs with intracranial activity and the typically late onset of BM. In the cohort of HR-positive/HER2-negative BC patients of a non-randomized phase 2 trial, the combination of the CDK4/6-inhibitor abemaciclib with ET yielded limited intracranial activity but most patients have already exhausted endocrine treatment options at the time of BM diagnosis [25]. Furthermore, recent RWD showed shorter CNS PFS and OS in patients receiving CDK4/6 inhibitors before and after BM development, when compared to those receiving CDK4/6 inhibitors only after BM development, thus postulating that prior CDK4/6 inhibitor exposure may lead to mechanisms of resistance impairing CDK4/6 inhibitor efficacy after BM occurrence [26]. Emerging evidence of CNS activity has also been reported for T-DXd in HER2-low disease (DEBBRAH trial) [27] and for the anti-HER3 ADC patritumab deruxtecan in the TUXEDO-3 trial [28,29].

More than 70% of patients in our cohort received some form of cranial radiotherapy. According to current EANO-ESMO guidelines, WBRT should be avoided in patients with up to 10 BM [2]. Detailed information on the number and location of BM was not available in our registry. Nevertheless, we observed a temporal decline in the use of WBRT as the primary local treatment modality. As expected, patients who underwent neurosurgical resection - typically representing cases with a limited number of BM - had the longest median OS of 21.9 months, followed by those who received focal radiotherapy (18.7 months). Patients treated with primary WBRT had the poorest outcomes, with a median OS of 6.2 months. These findings are consistent with prior studies [30].

A distinct subgroup of patients comprised those with brain-only metastatic disease, identified in 56 patients (11.7%) within our registry. In this subgroup, the rate of primary neurosurgical intervention was significantly higher compared to patients with concurrent extracranial metastases (38.0% vs. 6.9%; OR 8.0; p < 0.001). Moreover, OS following BM diagnosis was longer in these patients than in all other patients with BM (median OS 12.9 vs. 7.0 months; HR 0.58; 95%CI 0.42-0.80; p = 0.001), probably reflecting the additional relevance of extracranial disease status.

Importantly, retrospective data comparing outcomes between patients treated with neurosurgery or focal radiotherapy and those receiving WBRT have to be interpreted with caution, since patients treated with primary WBRT may have higher intracranial disease burden, more extensive disease, or a poorer performance status, which is known to influence survival outcomes independently of the treatment modality. A further limitation arises from the registry-based design: data on the clinical indication for (neurological symptoms vs. routine screening) and the imaging modality used for BM diagnosis (cranial CT vs. brain MRI) were not available, which may introduce ascertainment and lead-time bias affecting the observed incidence and survival estimates.

Notably, the receptor status of the BM was unknown in most cases, as biopsy or surgery was performed in only a minority of patients (15.7%). Among patients who underwent neurosurgical resection with available receptor data, changes in ER and HER2 status were observed in 18% (10 of 55) and 35.3% (18 of 51) of cases, respectively. The most common alterations were loss of ER expression and a gain of HER2 expression. Comparable rates of receptor status discordance have been reported by others [31,32] underscoring the challenge of selecting subtype-specific systemic therapy when the receptor status of brain metastases is unknown.

## 5 Conclusion

In this large real-world cohort of patients with MBC, 17.1% developed BM during the disease course, with the highest incidences observed among patients with HER2-positive (up to 35.3%) and triple-negative (28.5%) subtypes. The development of BM was associated with markedly reduced OS, particularly in patients with TNBC. Despite recent advances in as focal radiotherapy and CNS-active systemic agents, BM remain a major challenge across MBC subtypes and continue to drive poor outcomes, especially when BM are diagnosed late during the course of disease. Our findings highlight the need for improved prevention, earlier risk identification, and more effective subtype-adapted therapies, providing an essential foundation for future BM-focused clinical trials.

**Table:** Abbreviations

ADC	Antibody–drug conjugate
AGMT	Austrian Group for Medical Tumor Therapy
BBB	Blood–brain barrier
BC	Breast cancer
BM	Brain metastases
BMFS	Brain metastasis–free survival
BTB	Brain–tumor barrier
CDK4/6 inhibitor	Cyclin-dependent kinase 4/6 inhibitor
CI	Confidence interval
CNS	Central nervous system
DFS	Disease-free survival
EANO	European Association of Neuro-Oncology
ER	Estrogen receptor
ESMO	European Society for Medical Oncology
HER2	Human epidermal growth factor receptor 2
HR	Hazard ratio *or* Hormone receptor
LMD	Leptomeningeal disease
MBC	Metastatic breast cancer
mOS	Median overall survival
NST	No special type
OR	Odds ratio
OS	Overall survival
PARP inhibitor	Poly(ADP-ribose) polymerase inhibitor
QoL	Quality of life
RWD	Real-world data

## Ethics approval and consent to participate

The registry was approved by the Ethics Committee of the province Salzburg (IRB number: 415-E/1836). All patients provided written informed consent or died before data entry.

## Consent for publication

Not applicable.

## Availability of data and materials

The datasets used and/or analyzed during the current study are available from the corresponding author on reasonable request.

## Declaration of generative AI and AI-assisted technologies in the manuscript preparation process

During the preparation of this work, the authors used ChatGPT by OpenAI for language editing, grammatical correction, and refinement of scientific wording. After using this tool, the authors reviewed and edited the content as needed and take full responsibility for the content of the published article.

## Funding

Roche, Daiichi Sankyo, Novartis, Pfizer, Caris Life Science, Lilly, Seagen, Gilead, Stemline Therapeutics, and AstraZeneca.

## CRediT authorship contribution statement

**Simon Peter Gampenrieder:** Conceptualization, Data curation, Formal analysis, Writing – original draft, Writing – review & editing. **Vanessa Castagnaviz:** Data curation, Formal analysis, Writing – original draft, Writing – review & editing. **Rupert Bartsch:** Data curation, Formal analysis, Writing – review & editing. **Thamer Sliwa:** Data curation. **Angelika Pichler:** Data curation. **Renate Pusch:** Data curation. **Clemens Dormann:** Data curation. **Christoph Suppan:** Data curation. **Sonja Heibl:** Data curation. **Lukas Scagnetti:** Data curation. **Margit Sandholzer:** Data curation. **Thomas Winder:** Data curation. **August Felix Zabernigg:** Data curation. **Clemens Schmitt:** Data curation. **Daniel Egle:** Data curation. **Christopher Hager:** Data curation. **Petra Pichler:** Data curation. **Florian Roitner:** Data curation. **Johannes Andel:** Data curation. **Kathrin Strasser-Weippl:** Data curation. **Michael Hubalek:** Conceptualization. **Michael Knauer:** Conceptualization. **Christian Fridolin Singer:** Conceptualization, Data curation. **Richard Greil:** Conceptualization, Data curation, Funding acquisition, Supervision, Writing – review & editing. **Gabriel Rinnerthaler:** Conceptualization, Data curation, Formal analysis, Writing – original draft, Writing – review & editing.

## Declaration of competing interest

The authors declare the following financial interests/personal relationships which may be considered as potential competing interests:Simon Peter Gampenrieder reports financial support was provided by Roche, Daiichi Sankyo Inc, Seagen, Novartis, Bristol Myers Squibb Co., AstraZeneca Pharmaceuticals LP, Pfizer, Janssen-Cilag, Gilead, Eli Lilly and Company and Merck Sharp & Dohme Corp. Simon Peter Gampenrieder reports a relationship with Roche, Daiichi Sankyo Inc, Novartis, Pfizer, Caris Life Sciences, Eli Lilly and Company, Seagen, Gilead, AstraZeneca Pharmaceuticals LP and Stemline Therapeutics that includes: funding grants. Simon Peter Gampenrieder reports a relationship with Roche, Amgen, Novartis, Pfizer, Bayer, Celgene, Daiichi Sankyo Inc, Janssen-Cilag and Gilead that includes: travel reimbursement. Vanessa Castagnaviz reports financial support was provided by Roche, Daiichi Sankyo Inc, AstraZeneca Pharmaceuticals LP and Novartis. Vanessa Castagnaviz reports a relationship with Daiichi Sankyo Inc, Astra Zeneca, Gilead, Pierre Fabre, Eli Lilly and Company, Roche, Novartis and Stemline Therapeutics that includes: travel reimbursement. Rupert Bartsch reports financial support was provided by AstraZeneca Pharmaceuticals LP, Daiichi Sankyo Inc, Eisai, Eli Lilly and Company, Gilead, Grünenthal GMBH, Merck Sharp & Dohme Corp, Novartis, Pfizer, Pierre-Fabre, Roche and Stemline Therapeutics. Rupert Bartsch reports a relationship with AstraZeneca Pharmaceuticals LP, Daiichi Sankyo Inc and Merck Sharp & Dohme Corp that includes: funding grants. Rupert Bartsch reports a relationship with AstraZeneca Pharmaceuticals LP, Daiichi Sankyo Inc, Eli Lilly and Company, Gilead, Merck Sharp & Dohme Corp, Novartis, Pfizer and Roche that includes: travel reimbursement. Thamer Sliwa reports financial support was provided by Daiichi Sankyo Inc, Eli Lilly and Company, Gilead, Novartis, Pfizer and Roche. Angelika Pichler reports a relationship with Pierre Fabre, Janssen-Cilag, Sanofi, Eli Lilly and Company, Roche, BeiGene and AOP Orphan that includes: travel reimbursement. Renate Pusch reports financial support was provided by Roche, Novartis, Merck Sharp & Dohme Corp, AstraZeneca Pharmaceuticals LP, Stemline Therapeutics and Daiichi Sankyo Inc. Renate Pusch reports a relationship with Pfizer, AstraZeneca Pharmaceuticals LP, Gilead, Merck Sharp & Dohme Corp and Daiichi Sankyo Inc that includes: travel reimbursement. Clemens Dormann reports financial support was provided by Roche, Pfizer, Novartis, AstraZeneca Pharmaceuticals LP, Eli Lilly and Company, Merck Sharp & Dohme Corp, Gilead, Amgen, Sandoz, Daiichi Sankyo Inc and Stemline Therapeutics. Clemens Dormann reports a relationship with Roche, Pfizer, Novartis, AstraZeneca Pharmaceuticals LP, Merck Sharp & Dohme Corp, Sanofi and Daiichi Sankyo Inc that includes: travel reimbursement. Christoph Suppan reports financial support was provided by AstraZeneca Pharmaceuticals LP, Pfizer, Eli Lilly and Company, Novartis, Roche, Merck Sharp & Dohme Corp, Pierre Fabre, Daiichi Sankyo Inc, Gilead and Stemline Therapeutics. Sonja Heibl reports financial support was provided by Bristol Myers Squibb Co., Novartis, AstraZeneca Pharmaceuticals LP and Takeda. Sonja Heibl reports a relationship with Bristol Myers Squibb Co. and AstraZeneca Pharmaceuticals LP that includes: funding grants. Sonja Heibl reports a relationship with Roche, Sanofi, Abbvie and Pfizer that includes: travel reimbursement. Lukas Scagnetti reports financial support was provided by Novartis and Janssen-Cilag. Lukas Scagnetti reports a relationship with Celgene and AOP Orphan that includes: funding grants. Lukas Scagnetti reports a relationship with AstraZeneca Pharmaceuticals LP that includes: travel reimbursement. Margit Sandholzer reports financial support was provided by AstraZeneca Pharmaceuticals LP. Margit Sandholzer reports a relationship with Pfizer, Roche, Celgene, Eli Lilly and Company, Novartis and Merck Sharp & Dohme Corp that includes: travel reimbursement. Thomas Winder reports financial support was provided by Novartis, Roche, Bristol Myers Squibb Co., AstraZeneca Pharmaceuticals LP, Merck Sharp & Dohme Corp, Pfizer, Eli Lilly and Company, Daiichi Sankyo Inc, Janssen-Cilag, Gilead and Pierre-Fabre. Thomas Winder reports a relationship with Roche, Bristol Myers Squibb Co., AstraZeneca Pharmaceuticals LP, Daiichi Sankyo Inc, Pierre-Fabre and Merck Sharp & Dohme Corp that includes: travel reimbursement. August Felix Zabernigg reports financial support was provided by Roche, AstraZeneca Pharmaceuticals LP and Johnson & Johnson. August Felix Zabernigg reports a relationship with Roche and Eli Lilly and Company that includes: travel reimbursement. Clemens Schmitt reports financial support was provided by Roche and Janssen-Cilag. Clemens Schmitt reports a relationship with Roche and Janssen-Cilag that includes: funding grants. Clemens Schmitt reports a relationship with Roche, Janssen-Cilag, Takeda, Bristol Myers Squibb Co., AstraZeneca Pharmaceuticals LP and AbbVie that includes: travel reimbursement. Daniel Egle reports financial support was provided by Roche, Pfizer, Novartis, AstraZeneca Pharmaceuticals LP, Pierre Fabre, Eli Lilly and Company, Merck Sharp & Dohme Corp, Sirius Medical, Gilead, Daiichi Sankyo Inc and Menarini. Daniel Egle reports a relationship with Sirius Medical that includes: funding grants. Daniel Egle reports a relationship with Roche, Pfizer, Novartis, AstraZeneca Pharmaceuticals LP, Eli Lilly and Company, Merck Sharp & Dohme Corp, Sirius Medical, Gilead, Daiichi Sankyo Inc, Menarini that includes: travel reimbursement. Christopher Hager reports a relationship with Pfizer, Eli Lilly and Company, Roche and Novartis that includes: travel reimbursement. Petra Pichler reports a relationship with Kite, Roche, BeiGene and Janssen-Cilag that includes: travel reimbursement. Florian Roitner reports financial support was provided by Pierre Fabre, Pfizer, Roche, Gilead, Amgen, Abbvie, Janssen-Cilag, AOP Orphan, BeOne and Novartis. Florian Roitner reports a relationship with Pfizer, Eli Lilly and Company, Roche, Pierre Fabre, Merck Sharp & Dohme Corp, Amgen, Abbvie, BeOne, Sobi and Gilead that includes: travel reimbursement. Kathrin Strasser-Weippl reports financial support was provided by AstraZeneca Pharmaceuticals LP, Daiichi Sankyo Inc, Eli Lilly and Company, Gilead, Merck Sharp & Dohme Corp, Novartis, Pfizer, Roche and Seagen. Michael Hubalek reports financial support was provided by Amgen, Roche, Eli Lilly and Company, Pfizer, Novartis and Stemline Therapeutics. Michael Hubalek reports a relationship with Roche and Pfizer that includes: travel reimbursement. Michael Knauer reports a relationship with Agendia that includes: funding grants. Christian Fridolin Singer reports financial support was provided by Novartis, AstraZeneca Pharmaceuticals LP, Daiichi Sankyo Inc, Gilead and Stemline Therapeutics. Christian Fridolin Singer reports a relationship with Novartis, AstraZeneca Pharmaceuticals LP, Daiichi Sankyo Inc, Gilead and Stemline Therapeutics that includes: funding grants. Christian Fridolin Singer reports a relationship with Novartis, AstraZeneca Pharmaceuticals LP, Daiichi Sankyo Inc, Gilead and Stemline Therapeutics that includes: travel reimbursement. Richard Greil reports financial support was provided by Celgene, Roche, Merck, Takeda, AstraZeneca Pharmaceuticals LP, Novartis, Amgen, Bristol Myers Squibb Co., Merck Sharp & Dohme Corp, Sandoz and Abbvie. Richard Greil reports a relationship with Celgene, Merck, Takeda, AstraZeneca Pharmaceuticals LP, Novartis, Amgen, Bristol Myers Squibb Co., Merck Sharp & Dohme Corp, Sandoz, Gilead and Roche that includes: funding grants. Richard Greil reports a relationship with Roche, Amgen, Janssen-Cilag, AstraZeneca Pharmaceuticals LP, Novartis, Merck Sharp & Dohme Corp, Celgene, Gilead, Bristol Myers Squibb Co., Abbvie and Daiichi Sankyo Inc that includes: travel reimbursement. Gabriel Rinnerthaler reports financial support was provided by AstraZeneca Pharmaceuticals LP, Bristol Myers Squibb Co., Daiichi Sankyo Inc, Eli Lilly and Company, Gilead, Merck Sharp & Dohme Corp, Novartis, Seagen, and Stemline Therapeutics. Gabriel Rinnerthaler reports a relationship with AstraZeneca Pharmaceuticals LP, Daiichi Sankyo Inc, and Stemline Therapeutics that includes: funding grants. Gabriel Rinnerthaler reports a relationship with Daiichi Sankyo Inc, Gilead, Roche, and Servier that includes: travel reimbursement. If there are other authors, they declare that they have no known competing financial interests or personal relationships that could have appeared to influence the work reported in this paper.

## References

[1] Wang R. (2019). The clinicopathological features and survival outcomes of patients with different metastatic sites in stage IV breast cancer. BMC Cancer.

[2] Le Rhun E. (2021). EANO-ESMO clinical practice guidelines for diagnosis, treatment and follow-up of patients with brain metastasis from solid tumours. Ann Oncol.

[3] Arvanitis C.D., Ferraro G.B., Jain R.K. (2020). The blood-brain barrier and blood-tumour barrier in brain tumours and metastases. Nat Rev Cancer.

[4] Shah N. (2018). Investigational chemotherapy and novel pharmacokinetic mechanisms for the treatment of breast cancer brain metastases. Pharmacol Res.

[5] Pitz M.W. (2011). Tissue concentration of systemically administered antineoplastic agents in human brain tumors. J Neuro Oncol.

[6] Tsukada Y. (1983). Central nervous system metastasis from breast carcinoma. Autopsy study. Cancer.

[7] Kuksis M. (2021). The incidence of brain metastases among patients with metastatic breast cancer: a systematic review and meta-analysis. Neuro Oncol.

[8] Thulin A. (2020). Clinical outcome of patients with brain metastases from breast cancer - a population based study over 21 years. Breast.

[9] Graesslin O. (2010). Nomogram to predict subsequent brain metastasis in patients with metastatic breast cancer. J Clin Oncol.

[10] DiStefano A. (1979). The natural history of breast cancer patients with brain metastases. Cancer.

[11] Niwińska A., Murawska M., Pogoda K. (2010). Breast cancer brain metastases: differences in survival depending on biological subtype, RPA RTOG prognostic class and systemic treatment after whole-brain radiotherapy (WBRT). Ann Oncol.

[12] Sun M.S. (2022). Brain metastasis in de novo breast cancer: an updated population-level study from SEER database. Asian J Surg.

[13] Curigliano G. (2022). Tucatinib versus placebo added to trastuzumab and capecitabine for patients with pretreated HER2+ metastatic breast cancer with and without brain metastases (HER2CLIMB): final overall survival analysis. Ann Oncol.

[14] Bartsch R. (2024). Final outcome analysis from the phase II TUXEDO-1 trial of trastuzumab-deruxtecan in HER2-positive breast cancer patients with active brain metastases. Neuro Oncol.

[15] Harbeck N. (2024). Trastuzumab deruxtecan in HER2-positive advanced breast cancer with or without brain metastases: a phase 3b/4 trial. Nat Med.

[16] Gennari A. (2021). ESMO clinical practice guideline for the diagnosis, staging and treatment of patients with metastatic breast cancer. Ann Oncol.

[17] Geyer C.E. (2025). Survival with trastuzumab emtansine in residual HER2-Positive breast cancer. N Engl J Med.

[18] Pestalozzi B.C. (2013). CNS relapses in patients with HER2-positive early breast cancer who have and have not received adjuvant trastuzumab: a retrospective substudy of the HERA trial (BIG 1-01). Lancet Oncol.

[19] Pivot X. (2015). CEREBEL (EGF111438): a phase III, randomized, open-label study of lapatinib plus capecitabine versus trastuzumab plus capecitabine in patients with human epidermal growth factor receptor 2-Positive metastatic breast cancer. J Clin Oncol.

[20] Swain S.M. (2014). Incidence of central nervous system metastases in patients with HER2-positive metastatic breast cancer treated with pertuzumab, trastuzumab, and docetaxel: results from the randomized phase III study CLEOPATRA. Ann Oncol.

[21] Chan A. (2021). Final efficacy results of neratinib in HER2-positive hormone receptor-positive early-stage breast cancer from the phase III ExteNET trial. Clin Breast Cancer.

[22] Lin N.U. (2023). Tucatinib vs placebo, both in combination with trastuzumab and capecitabine, for previously treated ERBB2 (HER2)-positive metastatic breast cancer in patients with brain metastases: updated exploratory analysis of the HER2CLIMB randomized clinical trial. JAMA Oncol.

[23] Geyer C.E. (2025). Presented at ESMO Congress.

[24] Tutt A.N.J. (2021). Adjuvant olaparib for patients with BRCA1- or BRCA2-Mutated breast cancer. N Engl J Med.

[25] Tolaney S.M. (2020). A phase II study of abemaciclib in patients with brain metastases secondary to hormone receptor-positive breast cancer. Clin Cancer Res.

[26] Chew S.M. (2024). Impact of cyclin dependent kinase 4/6 inhibitors on breast cancer brain metastasis outcomes. Eur J Cancer.

[27] Vaz Batista M. (2024). Trastuzumab deruxtecan in patients with previously treated HER2-low advanced breast cancer and active brain metastases: the DEBBRAH trial. ESMO Open.

[28] Preusser M. (2025). Patritumab deruxtecan in leptomeningeal metastatic disease of solid tumors: the phase 2 TUXEDO-3 trial. Nat Med.

[29] Bartsch R. (2025). Patritumab deruxtecan (HER3-DXd) in patients with active brain metastases of breast cancer (TUXEDO-3): a multicentre, single-arm, phase 2 trial. Lancet Oncol.

[30] Jeene P.M. (2018). Survival after whole brain radiotherapy for brain metastases from lung cancer and breast cancer is poor in 6325 Dutch patients treated between 2000 and 2014. Acta Oncol.

[31] Timmer M. (2017). Discordance and conversion rates of Progesterone-, Estrogen-, and HER2/neu-Receptor status in primary breast cancer and brain metastasis mainly triggered by hormone therapy. Anticancer Res.

[32] Hulsbergen A.F.C. (2020). Subtype switching in breast cancer brain metastases: a multicenter analysis. Neuro Oncol.

