# Supplementary material for: Central nervous system metastases in metastatic breast cancer: Incidence, subtype-specific patterns, and outcomes from the Austrian AGMT_MBC registry
Source: Breast. 2026 Aug 3;89:104891. doi: 10.1016/j.breast.2026.104891 (PMC13486313; doi:10.1016/j.breast.2026.104891)
Supplement: Supplementary file 1 [file mmc1.docx]

**Central Nervous System Metastases in Metastatic Breast Cancer: Incidence, Subtype-Specific Patterns, and Outcomes from the Austrian AGMT_MBC Registry**

**SUPPLEMENT**

**Table S1.** Patient characteristics of patients with and without BM.

**Table S2.** Selected treatments for MBC in patients with and without BM.

**Table S3.** Incidence of BM in different breast cancer subtypes and different time periods, respectively. Only deceased patients were included (n = 1,877).

**Table S4.** Brain metastases-free survival (BMFS) = time between diagnosis of metastatic disease and detection of BM; median (95% Confidence interval).

**Table S5.** Multivariable, time-dependent OS analysis of patients with or without BM.

**Table S6.** Patient characteristics of patients with BM with and without extracranial metastases.

Table S7. Use of whole brain radiotherapy (WBRT) as primary local treatment, defined as start of radiotherapy within 2 months of BM diagnosis (regardless of prior focal radiotherapy or neurosurgery) according to the date of BM diagnosis and breast cancer subtype, respectively.

Figure S1. Overall survival after diagnosis of BM in patients with “brain only” versus concomitant extracranial metastases.

**Figure S2.** Discordance of receptor status between primary tumor and brain metastasis.

**Table S1.** Patient characteristics of patients with and without BM.

| **Characteristic** | **BM N = 477** | **no BM N = 2,298** | **p-value**^1^ |
| --- | --- | --- | --- |
| **Age** (median; range) | 57 (23-87) | 65 (22-97) | **<0.001** |
| **Molecular subtype** |  |  | **<0.001** |
| HR+/HER2- | 186 (39.0%) | 1,621 (70.5%) |  |
| HR+/HER2+ | 96 (20.1%) | 233 (10.1%) |  |
| HR-/HER2+ | 67 (14.0%) | 123 (5.4%) |  |
| TNBC | 128 (26.8%) | 321 (14.0%) |  |
| **ER status**^2^ |  |  | **<0.001** |
| Negative | 210 (44.0%) | 481 (20.9%) |  |
| Weak Positive | 32 (6.7%) | 85 (3.7%) |  |
| Average or strong positive | 235 (49.3%) | 1,732 (75.4%) |  |
| **HER2 status**^3^ |  |  | **<0.001** |
| 0 | 181 (38.1%) | 1,071 (46.9%) |  |
| Low | 131 (27.6%) | 856 (37.5%) |  |
| Positive | 163 (34.3%) | 356 (15.6%) |  |
| Unknown | 2 | 15 |  |
| **Histologic subtype** |  |  | **<0.001** |
| No special type (NST) | 358 (75.2%) | 1,559 (68.4%) |  |
| Invasive lobular carcinoma | 24 (5.0%) | 348 (15.3%) |  |
| Other subtypes | 95 (20.0%) | 371 (16.3%) |  |
| Unknown | 1 | 20 |  |
| **Disease-free survival (DFS)** |  |  | **<0.001** |
| *de novo* metastatic | 162 (35.8%) | 885 (41.0%) |  |
| DFS < 24 months | 131 (28.9%) | 367 (17.0%) |  |
| DFS ≥ 24 months | 160 (35.3%) | 908 (42.0%) |  |
| Unknown | 24 | 138 |  |
| **Extracranial visceral disease** | 251 (52.6%) | 994 (43.3%) | **<0.001** |
| **Leptomeningeal disease** | 55 (11.5%)^4^ | 70 (3.0%) | **<0.001** |
| **Number of metastatic sites** |  |  | **<0.001** |
| 1 | 232 (48.6%) | 1,227 (53.4%) |  |
| 2-3 | 179 (37.5%) | 914 (39.8%) |  |
| ≥4 | 66 (13.8%) | 157 (6.8%) |  |
| **Survival status** |  |  | **<0.001** |
| Alive | 60 (12.6%) | 747 (32.5%) |  |
| Dead | 404 (84.7%) | 1,473 (64.1%) |  |
| Lost to follow-up | 13 (2.7%) | 78 (3.4%) |  |
| *^1^ Wilcoxon rank sum test; Pearson’s Chi-squared test*  *^2^ ER negative was defined as <1% positive tumor cells, an immune-reactive score (IRS) of 0-1 or a Allred score of 0-2; ER weak positive was defined as 1-9% positive tumor cells, an IRS of 2-3 or a Allred score of 3-4; ER average or strong positive was defined as 10-100% positive tumor cells, an IRS of 4-12 or a Allred score of 5-8.*  *^3^ HER2 negative was divided into HER2-0, and HER2-low (defined as immunohistochemically 1+ or 2+ without HER2 amplification)* | | | |
| *^4^ 33 (60.0%) diagnosed after diagnosis of BM, 8 (14.5%) diagnosed before diagnosis of BM, 14 (25.5%) diagnosed simultaneously with BM* | | | |

**Table S2.** Selected treatments for MBC in patients with and without BM.

|  | **BM (N = 477)** | | | | **no BM (N = 2,298)** | | | |
| --- | --- | --- | --- | --- | --- | --- | --- | --- |
|  | **HR+/HER2-** N = 186 | **HR+/HER2+** N = 96 | **HR-/HER2+** N = 67 | **TNBC** N = 128 | **HR+/HER2-** N = 1,621 | **HR+/HER2+** N = 233 | **HR-/HER2+** N = 123 | **TNBC** N = 321 |
| Median number of total treatment lines (range) | 3  (0-14) | 3  (0-10) | 2  (0-8) | 2  (0-9) | 2  (0-14) | 2  (0-13) | 1  (0-10) | 1  (0-8) |
| Median number of lines before diagnosis of BM (range) | 1  (0-8) | 1  (0-8) | 1  (0-5) | 1  (0-7) | NA | NA | NA | NA |
| CDK4/6 inhibitor | 90  (**48.4%**) | 7  (7.3%) | 0  (0.0%) | 1  (0.8%) | 975  (**60.1%**) | 34  (14.6%) | 1  (0.8%) | 10  (3.1%) |
| Trastuzumab emtansine (T-DM1) | 3  (1.6%) | 37  (**38.5%**) | 21 (**31.3%**) | 1  (0.8%) | 16  (1.0%) | 51  (**21.9%**) | 22  (**17.9%**) | 1  (0.3%) |
| Trastuzumab deruxtecan (T-DXd) | 19 (10.2%) | 18  (**18.8%**) | 10 (**14.9%**) | 4  (3.1%) | 89  (5.5%) | 36  (**15.5%**) | 13  (**10.6%**) | 8  (2.5%) |
| Lapatinib/Neratinib/Tucatinib | 2  (1.1%) | 44  (**45.8%**) | 40 (**59.7%**) | 1  (0.8%) | 9  (0.6%) | 36  (**15.5%**) | 27  (**22.0%**) | 2  (0.6%) |
| Trastuzumab/Pertuzumab | 6  (3.2%) | 83  (**86.5%**) | 54 (**80.6%**) | 4  (3.1%) | 41  (2.5%) | 177 (**76.0%**) | 102 (**82.9%**) | 5  (1.6%) |
| PARP inhibitor | 7  (**3.8%**) | 0  (0.0%) | 0  (0.0%) | 11 (**8.6%**) | 30  (**1.9%**) | 3  (1.3%) | 0  (0.0%) | 8  (**2.5%**) |
| Checkpointinhibitor | 3  (1.6%) | 1  (1.0%) | 1  (1.5%) | 17  (**13.3%**) | 17  (1.0%) | 1  (0.4%) | 0  (0.0%) | 44  (**13.7%**) |

**Table S3.** 5-year incidence* of BM in different breast cancer subtypes and different time periods, respectively.

| **Time interval** | **All** | **HR+/HER2-** | **HR+/HER2+** | **HR-/HER2+** | **TNBC** |
| --- | --- | --- | --- | --- | --- |
| 2005-2011 | **7.9%** | **5.7%** | **7.0%** | **20.0%** | **8.4%** |
| 2012-2018 | **11.1%** | **4.6%** | **14.7%** | **18.3%** | **29.0%** |
| 2019-2025 | **10.3%** | **6.1%** | **15.2%** | **21.9%** | **22.3%** |

Legend: 5-year incidence = 5y cumulative incidence plus baseline incidence of BM.

**Table S4.** Brain metastases-free survival (BMFS) = time between diagnosis of metastatic disease and detection of BM; median (95% Confidence interval).

| **Time interval** | **All** | **HR+/HER2-** | **HR+/HER2+** | **HR-/HER2+** | **TNBC** |
| --- | --- | --- | --- | --- | --- |
| 2005-2025 | N = 477  **12.4** months  (95%CI 11.0-14.8) | N = 186  **17.9** months  (95% CI 12.9–22.3) | N = 96  **16.4** months  (95% CI 12.3–25.5) | N = 67  **11.0** months  (95% CI 9.3–17.3) | N = 128  **7.1** months  (95% CI 0.0–9.6) |

**Table S5.** Multivariable, time-dependent OS analysis of patients with or without BM.

| **Risk for death** | **HR (95%-CI)** | ***p*** |
| --- | --- | --- |
| **Brain metastasis** *yes vs. no* | 4.23 (3.73-4.81) | **<0.001** |
| **Breast cancer subtype**  *HR-/HER2+ vs. HR+/HER2-*  *HR+/HER2+ vs. HR+/HER2-*  *TNBC vs. HR+/HER2-* | 0.77 (0.63-0.94)  0.63 (0.54-0.74)  2.01 (1.77-2.30) | **0.027**  **<0.001**  **<0.001** |
| **Disease-free survival (DFS)**  *≥ 24 months or de novo vs. < 24 months* | 0.63 (0.56-0.71) | **<0.001** |
| **Extracranial visceral disease***  *yes vs. no* | 0.97 (0.87-1.08) | 0.534 |
| **Number of metastatic sites***  *1 vs. ≥ 4*  *2-3 vs. ≥ 4* | 0.55 (0.46-0.67)  0.80 (0.67-0.95) | **<0.001**  **0.027** |
| **Age***  *Premenopausal*  *Postmenopausal* | 1.03 (1.02-1.04)  1.03 (1.02-1.03) | **<0.001**  **<0.001** |

* at diagnosis of metastatic disease

**Table S6.** Patient characteristics of patients with BM with and without extracranial metastases.

| **Characteristic** | **BM and extracranial metastases** N = 421 | **BM only** N = 56 | **p-value***^1^* |
| --- | --- | --- | --- |
| **Age** (median; range) | 58 (23-87) | 55 (27-85) | 0.300 |
| **Molecular subtype** |  |  | 0.140 |
| HR+/HER2- | 170 (40.4%) | 16 (28.6%) |  |
| HR+/HER2+ | 84 (20.0%) | 12 (21.4%) |  |
| HR-/HER2+ | 54 (12.8%) | 13 (23.2%) |  |
| TNBC | 113 (26.8%) | 15 (26.8%) |  |
| **Disease-free survival (DFS)** |  |  | **<0.001** |
| *de novo* metastatic | 159 (39.8%) | 3 (5.6%) |  |
| DFS < 24 months | 96 (24.1%) | 35 (64.8%) |  |
| DFS ≥ 24 months | 144 (36.1%) | 16 (29.6%) |  |
| Unknown | 22 | 2 |  |
| **Extracranial visceral disease** | 251 (59.6%) | - | **<0.001** |
| **Leptomeningeal disease** | 48 (11.4%) | 7 (12.5%) | 0.800 |
| **Number of metastatic sites** |  |  | **<0.001** |
| 1 | 176 (41.8%) | 56 (100.0%) |  |
| 2-3 | 179 (42.5%) | - |  |
| ≥4 | 66 (15.7%) | - |  |
| **Local therapy** |  |  | **<0.001** |
| Neurosurgery | 29 (6.9%) | 21 (37.5%) |  |
| Focal radiotherapy | 53 (12.6%) | 4 (7.1%) |  |
| Whole brain radiotherapy (WBRT) | 173 (41.1%) | 21 (37.5%) |  |
| Other | 166 (39.4%) | 10 (17.9%) |  |
| **Median number of lines before diagnosis of BM** | 1.00 (0.00-8.00) | - | **<0.001** |
| *^1^Wilcoxon rank sum test; Fisher‘s exact test, chi-square test;* | | | |

Table S7. Use of whole brain radiotherapy (WBRT) as primary local treatment, defined as start of radiotherapy within 2 months of BM diagnosis (regardless of prior focal radiotherapy or neurosurgery) according to the date of BM diagnosis and breast cancer subtype, respectively.

| **Time interval** | **All** | **HR+/HER2-** | **HR+/HER2+** | **HR-/HER2+** | **TNBC** |
| --- | --- | --- | --- | --- | --- |
| 2005-2025 | 194/477  (**40.7**%) | 69/186  (**37.0**%) | 41/96  (**41.7**%) | 25/67  (**36.2**%) | 59/128  (**46.0**%) |
| 2005-2011 | 43/78  (**55.1%**) | 13/27  (**48.1%**) | 13/21  (**61.9%**) | 12/18  (**66.7%**) | 5/12  (**41.7%**) |
| 2012-2018 | 99/219  (**45.2%**) | 35/81  (**43.2%**) | 16/38  (**42.1%**) | 9/29  (**31.0%**) | 39/71  (**54.9%**) |
| 2019-2025 | 52/180  (**28.9%**) | 21/78  (**26.9%**) | 12/37  (**32.4%**) | 4/20  (**20.0%**) | 15/45  (**33.3%**) |

Figure S1: Univariable OS analysis of patients with brain-only disease versus patients presenting with BM and concurrent extracranial metastases.

**Figure S2.** Discordance of receptor status between primary tumor and brain metastasis.

**Legend:** Sankey diagrams of ER (A), HER2 status (B) and breast cancer subtype (C) from the latest primary tumor (left) and first BM (right), respectively.
